# Proceedings of the Annual Meeting of the Æsculapian Society, Held at Charleston, November 28th and 29th, 1855

**Published:** 1856-01

**Authors:** S. York, C. M. Hamilton

**Affiliations:** President; Secretary


ARTICLE IV.—*Proceedings of the Annual Meeting of the Æsculapian Society, held at Charleston, November 28th and 29th*, 1855.

The Meeting was called to order by the President, Dr. T. D. Washburn.

The Secretary being absent, Dr. C. Hamilton was appointed Secretary *pro tem.*

The proceedings of the last meeting were read and adopted.

The roll of Members being called, about twenty were found in attendance.

Two of the Censors being absent, the President appointed Drs. D. W. Stormont and E. C. Banks, Censors *pro tem.*

The Censors retired to receive applications for membership.

On motion of Dr. York, ordered that the Secretary notify those members, who have been absent from the meetings of the society for the last two years, that unless they attend the next meeting, and fill the requirements of the by-laws, they will be considered as no longer members of the society.

The Censors came in and reported favorably on the application of the following gentlemen:

Drs. H. J. E. Balch, of Georgetown; J. W. Cameron, and —— Chambers of Charleston; and J. Y. Hitt, of Sullivan.

Whereupon the Society proceeded to ballot on their application and they were unanimously elected.

On motion of Dr. Banks, ordered that any member who shall divulge the name of a person who shall apply for membership, and be rejected, shall be fined in the sum of five dollars for each offence.

The reading of essays being in order, Dr. Davis, of Paris, read a lengthy and interesting essay, on the cause and treatment of epidemic dysentery, relating several cases which occured in his practice.

This essay elicited a spirited discussion, which was participated in by many of the members.

Dr. York gave an historical account of the same disease as it prevailed in and around Paris, reporting orally the treatment of several cases.

EVENING SESSION, 7 O'CLOCK, P. M.

Society met at the Court House, where a large assembly had collected to hear the public addresses. Dr. York, V. P., in the chair, called to order, and introduced to the audience, Dr. D. W. Stormont, who gave a very spirited and interesting address, on the subject of medical societies.

After which, the President delivered his valedictory address, which elicited the applause of the audience.

The audience was then dismissed, and the society called to order by the President.

An interesting case was reported by Dr. Richmond, the treatment of which was freely discussed.

On motion of Dr. York, the society then proceeded to the election of officers for the ensuing year.

The following gentlemen were unanimously elected to the offices attached to their respective names:

| | | | |
|---|---|---|---|
| Dr. S. York | *President.* | Dr. E. C. Banks | *Censors.* |
| ,, D. W. Stormont | *Vice President.* | ,, —— Chambers | |
| ,, C. M. Hamilton | *Secretary.* | ,, John Tenbrook | |
| ,, T. D. Washburn | *Treasurer.* | ,, F. R. Payne, and C. Johnson | |

On motion, adjourned to meet at the Court House to-morrow morning, at 9 o'clock, A. M.

THURSDAY, 9 O'CLOCK, A. M., NOVEMBER 29TH.

Society met as per adjournment, and was called to order by the President.

On motion of Dr. Washburn, it was ordered that the President, Vice President, and Secretary, be, and they are hereby constituted *ex-officio* a publishing committee.

On motion, the Secretary was ordered to procure such books and stationery as may be necessary for the purposes of his office.

On motion of Dr. McClure, the Secretary was directed to inform all members who are in arrears, of the amount of their indebtedness, and request immediate payment.

On motion, the society proceeded to elect delegates to the State and National Association, which resulted as follows:

Drs. S. York, E. C. Banks, John Tenbrook, and W. B. Duffield, to the National Association, with power to appoint substitutes.

Drs. James H. Steel, A. McClure, J. W. Cameron, and T.

D. Washburn, to the State Medical Society, with power also to appoint substitutes.

On motion of Dr. Davis, ordered that the next meeting of this society be held at York, on the last Wednesday in May next.

Dr. Richmond, chairman of a committee appointed at the last meeting of this society, to prepare a bill and memorial to the State Legislature, praying for an act to legalize the dissection of dead human subjects, and other purposes, made a lengthy report, which was ordered to be printed.

On motion of Dr. Mitchell, our delegates to the State Medical Society, were instructed to obtain the concurrent action of said society with ours in procuring the above action of the Legislature.

On motion of Dr. Chambers, ordered, that our delegates to the State Medical Society, be instructed to urge upon said society, the importance of taking steps to secure the passage of a Registration Law.

On motion of Dr. Banks, resolved, that this society unanimously request of Dr. Stormont, a copy of his public address for publication.

Resolved, that this society unanimously request of Dr. Washburn, a copy of his valedictory address for publication.

In conformity with the constitution, the President made the following appointments for the next meeting of the society.

Drs. Washburn and Chambers to read essays on substitutes for Quinine. Drs. Hitt and Balch to read essays on subjects of their own selection.

The following members were appointed chairmen of a committee of their own selection, and to report on the subjects annexed to their several names.

| | | | |
|---|---|---|---|
| Dr. | E. C. Banks | on | Surgery. |
| ,, | F. R. Payne | ,, | Practical Medicine. |
| ,, | —— Chambers | ,, | Epidemics. |
| ,, | John Tenbrook | ,, | Midwifery. |
| ,, | J. M. Logan | ,, | Indigenous Botany. |
| ,, | D. W. Stormont | ,, | Chol. Infantum. |
| ,, | A. W. McClure | ,, | Topography of Wabash Valley. |

| | | | |
|---|---|---|---|
| ,, | D. O. McCord | ,, | Intermittent Fever. |
| ,, | H. W. Davis | ,, | Pneumonia. |
| ,, | J. S. Richmond | ,, | Typhoid Fever. |
| ,, | C. M. Hamilton | ,, | Prolapsus Uteri. |

On motion of Dr. Washburn, Resolved, that the thanks of this society are tendered to the members of the profession in Charleston for their generous accommodation and kind attentions during their present session.

Resolved, that at each annual meeting this society may elect two honorary members who shall not reside within its territorial limits at the time of the election.

On motion of Dr. Washburn, Dr. —— Clippinger, of Terre Haute, Indiana, was unanimously elected an honorary member of this society.

On motion of Dr. Davis, Dr. E. Reed, of Terre Haute, was unanimously elected an honorary member of this society.

On motion of Dr. Banks, Resolved, that the thanks of this society are due to Dr. T. D. Washburn for the able and impartial manner in which he has discharged the duties of President, during the last year.

On motion, the Secretary was instructed to forward a copy of the proceedings of this meeting for publication in the N. W. Medical and Surgical Journal. Also, that he furnish a copy to the editor of the Charleston Courier, requesting the editors of the Paris and Marshall papers, Greenup Tribune, and Vincennes Gazette, and all papers desirous of promoting the interests of our society, to publish the same.

On motion, society adjourned to meet at York, Clark Co., on the last Wednesday of May next.

S. York, M. D., *President.*

C. M. Hamilton, M. D., *Secretary.*

